# Road pedestrian detection and tracking algorithm based on improved YOLOv5s and DeepSORT

**DOI:** 10.1371/journal.pone.0334786

**Published:** 2025-11-04

**Authors:** Guofeng Qin, Rongting Pan, Yi Deng, Peiwen Mi, Yongjian Zhu

**Affiliations:** 1 Teachers College for Vocational and Technical Education, Guangxi Normal University, Guilin, China; 2 School of Information Engineering, Guangxi Vocational College of Water Resources and Electric Power, Nanning, China; 3 Guangxi Key Laboratory of Brain-inspired Computing and Intelligent Chips, School of Electronic and Information Engineering, Guangxi Normal University, Guilin, China; 4 Guilin Normal University, Guilin, China; 5 College of Engineering Physics, Shenzhen Technology University, Shenzhen, China; G H Raisoni College of Engineering and Management, Pune, INDIA

## Abstract

To address the challenges of low accuracy, high miss detection rate, and poor tracking stability in pedestrian detection and tracking under dense occlusion and small object scenarios on traffic roads, this paper proposes a pedestrian detection and tracking algorithm based on improved YOLOv5s and DeepSORT. For the improvements in the YOLOv5s detection network, first, the Focal-EIoU loss function is used to replace the CIoU loss function. Second, a 160 × 160-pixel Small Object (SO) detection layer is added to the Neck structure. Finally, the Multi-Head Self-Attention (MHSA) mechanism is introduced into the Backbone network to enhance the model’s detection performance. Regarding the improvements in the DeepSORT tracking framework, a lightweight ShuffleNetV2 network is integrated into the appearance feature extraction network, reducing the number of model parameters while maintaining accuracy. Experimental results show that the improved YOLOv5s achieves an mAP0.5 of 80.8% and an mAP0.5:0.95 of 49.7%, representing increases of 4.4% and 3.9%, respectively, compared to the original YOLOv5s. The enhanced YOLOv5s-DeepSORT achieves an MOTA of 50.7% and an MOTP of 77.3%, improving by 3.3% and 0.5%, respectively, over the original YOLOv5s-DeepSORT. Additionally, the number of identity switches (IDs) is reduced by 11.3%, and the model size is reduced to 20% of the original algorithm, enhancing its portability. The proposed method demonstrates strong robustness and can effectively track targets of different sizes.

## 1. Introduction

With the increase in urban population and traffic volume, pedestrian detection and tracking have become increasingly important in the fields of intelligent transportation and public safety. Accurate and efficient pedestrian detection and tracking contribute to pedestrian behavior analysis, improving the efficiency and reliability of road safety monitoring and Intelligent Transportation Systems [1] (ITS). In pedestrian traffic management [2], real-time detection and tracking of pedestrians in monitored areas assist security personnel in promptly identifying abnormal behaviors or potential threats. In ITS, pedestrian detection and tracking can be applied to traffic flow statistics and accident early warning, providing crucial support for urban traffic management. Additionally, in the field of autonomous driving [3], accurately perceiving and tracking pedestrians on the road is one of the core technologies for ensuring driving safety, enabling autonomous driving systems to make reasonable decisions based on pedestrian behavior. Therefore, pedestrian detection and tracking have become one of the key research areas in computer vision and intelligent transportation.

Traditional object detection methods typically rely on manually designed features [4] and perform object recognition through multiple independent steps. However, they involve high computational costs and slow detection speeds, making it difficult to meet the requirements of real-time object detection. The development of deep learning has significantly advanced object detection technology. YOLOv5s is a deep-learning-based object detection algorithm that achieves end-to-end training through a multi-scale feature fusion strategy, enabling simultaneous optimization of bounding box prediction and category recognition. Compared with traditional methods, YOLOv5s has significantly improved both feature representation ability and computational efficiency, allowing it to perform object detection at a relatively high frame rate and meet real-time detection requirements. However, its detection accuracy still has certain limitations in complex scenarios such as low-light conditions, dense occlusion, and small pedestrian targets.

Currently, multi-object tracking (MOT) methods are mainly categorized into Tracking-by-Detection [5] (TBD) and Joint Detection and Embedding [6] (JDE) methods. Among these, detection-based tracking is the mainstream approach. This method first utilizes an object detection algorithm to identify target regions in video frames and then matches detection boxes belonging to the same target through an association model to construct a complete motion trajectory. However, the tracking performance of such methods largely depends on the quality of target feature extraction. As a widely used detection-based tracking algorithm, DeepSORT [7] has significantly improved in terms of stability and robustness compared to SORT [8]. However, it still has certain limitations in practical applications. Firstly, its appearance feature extraction network employs a relatively complex deep neural network, resulting in high computational overhead, which limits its application in real-time pedestrian tracking tasks. When running on resource-constrained edge devices, it may lead to reduced processing efficiency. Secondly, in scenarios involving dense pedestrians, severe occlusion, or small targets, the target association robustness of DeepSORT decreases, making identity switches (IDs) more likely to occur, thereby affecting tracking stability and accuracy. Therefore, optimizing the pedestrian appearance feature extraction network of DeepSORT to enhance feature representation ability while reducing computational complexity will contribute to improving overall tracking performance.

To address the above issues, this paper proposes a pedestrian detection and tracking algorithm based on an improved YOLOv5s-DeepSORT, optimizing both the detection network and the feature extraction network. This method employs an improved YOLOv5s as the pedestrian detector and inputs the detection results into the DeepSORT algorithm to achieve end-to-end pedestrian tracking. The optimized model enhances lightweight characteristics while maintaining excellent tracking performance, making it easier to deploy on edge devices. The main contributions of this paper are as follows:

(1) The original CIoU loss function in YOLOv5s is replaced with the Focal-EIoU loss function to improve the localization accuracy of bounding box regression, thereby enhancing pedestrian detection accuracy.
(2) A Small Object (SO) detection layer is added to the Neck structure of YOLOv5s, introducing a 160 × 160 detection feature map for detecting pedestrian targets larger than 4 × 4 in size, thereby improving the model’s ability to detect small-sized pedestrians.
(3) The Multi-Head Self-Attention (MHSA) mechanism is integrated into the backbone network of YOLOv5s, enabling the model to fully capture global contextual information and enhancing pedestrian detection performance and robustness.
(4) In the appearance feature extraction network of DeepSORT, the original deep neural network is replaced with the lightweight ShuffleNetV2 network, and the appearance feature extraction model is retrained. While maintaining good accuracy, this effectively reduces model parameters and computational complexity.

The remainder of this paper is organized as follows. Section 2 introduces related work on pedestrian detection and tracking. Section 3 elaborates on our improvements to the pedestrian detection network and tracking algorithm. Section 4 analyzes the experimental results. Finally, Section 5 concludes this paper and discusses future research directions.

## 2. Related work

Pedestrian detection and tracking technology based on deep learning leverages the fundamental principles and methods of computer vision, utilizing deep learning algorithms to detect and track pedestrian targets.

### 2.1. Object detection

In recent years, deep learning models represented by convolutional neural networks (CNN) [5] have achieved significant progress in the field of pedestrian detection. For example, object detection models such as the R-CNN series [9–11], SSD [12], and the YOLO series [13–15] have demonstrated excellent performance. Zhang et al. [16] incorporated a cross-channel attention mechanism into the Faster R-CNN network structure, improving the localization accuracy of bounding box regression and thereby enhancing the detection accuracy of occluded pedestrians. Li et al. [17] improved the SSD algorithm by introducing a Feature Pyramid Network (FPN) and proposed the Feature Fusion SSD (FSSD) algorithm, which improved detection accuracy. Yin et al. [18] proposed an improved YOLOv5 algorithm that enhances long-term attention and dependencies in image processing by integrating a large-kernel attention module and the C3 module. Additionally, they optimized the loss function, leading to improved pedestrian detection accuracy in road scenes. Zhu et al. [14] introduced the YOLOv7 algorithm, which significantly enhances object detection accuracy through a dynamic label assignment strategy and an extended efficient layer aggregation network, without increasing inference costs. Dou et al. [19] proposed an improved YOLOv8 algorithm, which integrates a multi-scale feature fusion mechanism and an optimized non-maximum suppression (NMS) algorithm, effectively reducing duplicate detections and missed detections in pedestrian detection.

### 2.2. Multi-object tracking

Bewley et al. [8] proposed the SORT algorithm, a lightweight multi-object tracking method based on Kalman filtering and the Hungarian algorithm. It utilizes detection results provided by an object detector for inter-frame object matching, enabling efficient online tracking. Wojke et al. [7] introduced the DeepSORT algorithm, which extends SORT by incorporating person re-identification (ReID) technology as an appearance model. It also employs a cascade matching strategy that prioritizes high-confidence targets, thereby improving matching accuracy and tracking stability. Wang et al. [20] proposed the JDE algorithm, which integrates object detection and person re-identification (ReID) feature extraction within a single neural network. This unified approach reduces inference time and enhances the real-time performance of multi-object tracking. Zhang et al. [21] introduced the YOLOX detector and the ByteTrack tracker. ByteTrack improves the detection result filtering strategy by retaining high-confidence detections while re-matching low-confidence detections in subsequent frames. This approach reduces missed detections and enhances the continuity of object trajectories. Hu et al. [22] proposed an algorithm based on an improved SSD and DeepSort, which enhances pedestrian detection capability in low-visibility scenarios by optimizing the SSD model and achieves stable pedestrian tracking under complex nighttime interference using DeepSort. Zhang et al. [23] proposed a pedestrian tracking algorithm designed to address occlusion issues. This algorithm integrates a dual-path self-attention mechanism and a cyclic-shift negative sample generation strategy. Additionally, it employs the least squares algorithm and Kalman filtering to predict the trajectories of tracked targets, thereby improving pedestrian tracking accuracy in occluded scenarios.

### 2.3. Object detection and tracking

The object detector can provide the initial position information of the target [24], defining the starting point for the tracker. Multi-object tracking [25] focuses on analyzing the motion trajectory of targets over time, closely intertwining with and complementing the object detection task. Through collaborative integration, these two tasks leverage each other’s strengths, enhancing the system’s robustness and real-time performance, making it well-suited for pedestrian tracking tasks in various complex scenarios.

Deep learning-based pedestrian detection and tracking algorithms are increasingly applied in traffic scenarios and have achieved remarkable success. However, numerous challenges remain in different pedestrian environments, such as low-light conditions, high pedestrian density, occlusion, and small pedestrian targets, which lead to high false detection and missed detection rates. Additionally, achieving a comprehensive optimization of the algorithm to balance accuracy, real-time performance, and lightweight deployment for practical applications remains an ongoing challenge. To address these challenges, we employ an improved YOLOv5s-DeepSORT algorithm for detecting and tracking densely occluded pedestrians.

## 3. Proposed methods

With the continuous optimization of object detection performance, detection-based tracking methods have been driven to develop in the field of multi-object tracking and have achieved significant results in pedestrian detection and tracking.

### 3.1. The YOLOv5s pedestrian detection model

**Fig 1:**
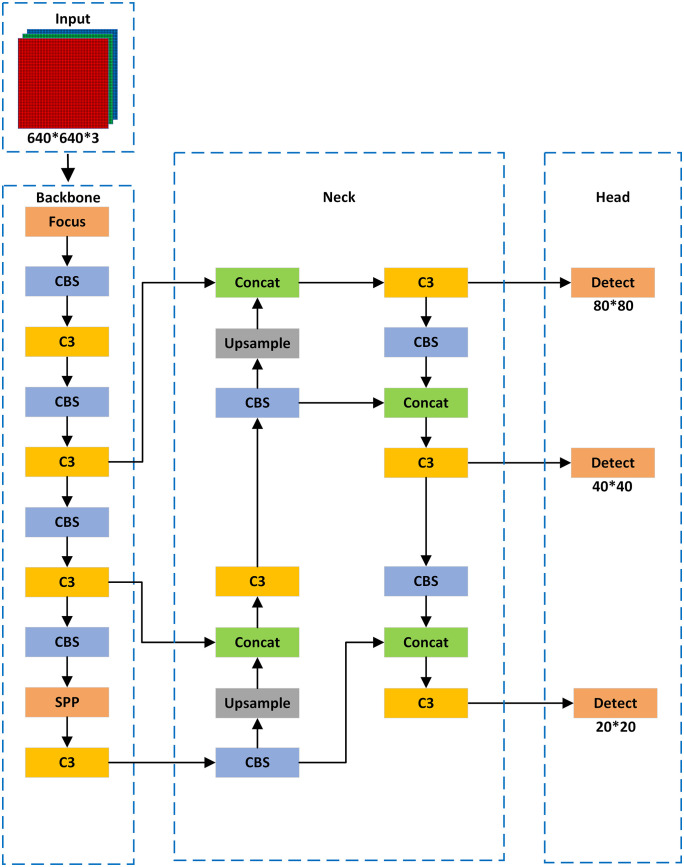
YOLOv5s network structure.

**Fig 2:**
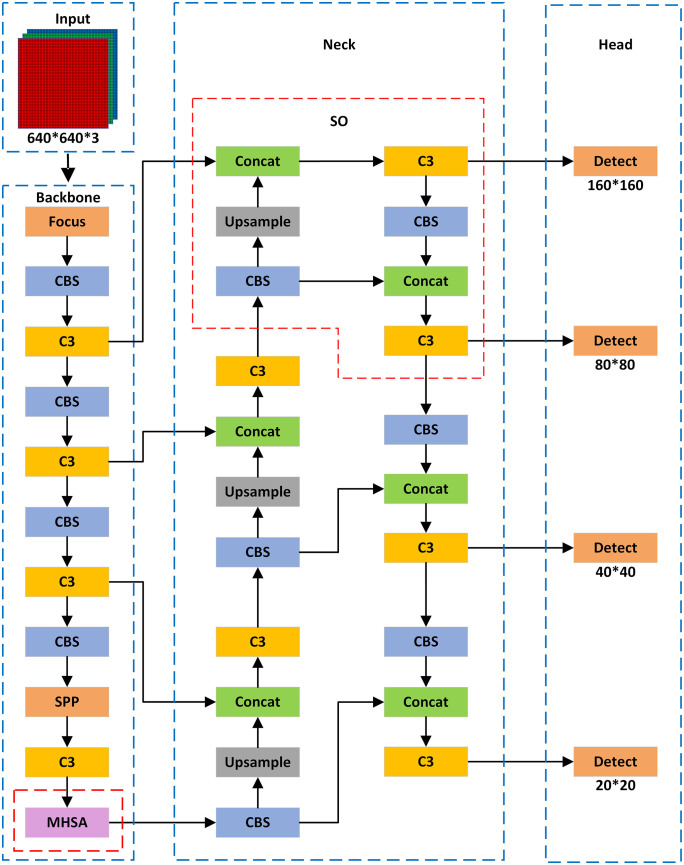
Improved YOLOv5s network structure.

The YOLOv5 model is a real-time and accurate algorithm widely used in the field of object detection. Based on the size of the model, it is divided into variants such as s, m, l, and x, with each variant differing in network depth and width. Although the overall average precision of YOLOv5s may be relatively lower, it has fewer parameters and lower computational complexity, achieving a good balance between accuracy and efficiency. Therefore, in this study, we adopted the YOLOv5s model, as shown in Fig 1, with the improved YOLOv5s model shown in Fig 2.

The YOLOv5s network consists of four components: the input stage (Input), the backbone network (Backbone), the neck structure (Neck), and the detection network (Head). The input stage is responsible for tasks such as Mosaic data augmentation, adaptive anchor box calculation, and adaptive image scaling. The backbone is used to extract features from the image while continuously reducing the size of the feature maps. The CBS module, consisting of a 2D convolutional layer (Conv), batch normalization layer (BN), and the SiLU (Sigmoid-Weighted Linear Unit) activation function, is a commonly used module in convolutional neural networks. The detection layer is located between the backbone and the head structure, combining the SPP (Spatial Pyramid Pooling) module and the FPN [26] (Feature Pyramid Network) with a PAN [27] (Path Aggregation Network) module. This design helps to fuse features of different scales, enhancing detection performance for multi-scale object features. The detection network is responsible for predicting image features, generating bounding boxes, and predicting classes, recording the classification and location information of the objects.

#### 3.1.1. Loss function improvement.

YOLOv5s employs the CIoU [28] (Complete Intersection over Union) loss function to compute the localization error between the target box and the predicted box. The calculation formula for CIoU is as follows:


$$\begin{array}{c} L_{CIoU}\;=1-IoU+\frac{\rho^{\text{2}}(b,{\;b}^{gt})}{c^{\text{2}}}+\alpha v \end{array}$$ (1)

$$IoU=\frac{A\cap B}{A\cup B}$$ (2)

$$\alpha =\frac{v}{(1-IoU)+v}$$ (3)

$$\begin{array}{c} v=\frac{\text{4}}{\pi^{2}}{\left(\arctan\frac{w^{gt}}{h^{gt}}-\arctan\frac{w}{h}\right)}^{2} \end{array}$$ (4)

$$\begin{array}{c} \frac{\partial v}{\partial w}=\frac{\text{8}}{\pi^{2}}\left(\arctan\frac{w^{gt}}{h^{gt}}-\arctan\frac{w}{h}\right)\times \frac{h}{w^{2}+h^{2}} \end{array}$$ (5)

$$\begin{array}{c} \frac{\partial v}{\partial h}=-\frac{\text{8}}{\pi^{2}}\left(\arctan\frac{w^{gt}}{h^{gt}}-\arctan\frac{w}{h}\right)\times \frac{w}{w^{2}+h^{2}} \end{array}$$ (6)

The EIoU [29] (Efficient Intersection over Union) loss function improves upon the shortcomings of the CIoU loss function by dividing the loss into three components: distance loss, direction loss, and IoU (Intersection over Union) loss. The parameters *α* and *v* of CIoU are modified, where *Cw* and *Ch* represent the length and height of the smallest box covering the ground truth box and anchor box. This addresses the issues caused by CIoU using aspect ratios. By integrating the EIoU loss function and the FocalL1 [29] loss function, the final Focal-EIoU [29] loss function is obtained.

To enhance model performance and accuracy, this experiment adopts the Focal-EIoU loss function to replace the original CIoU loss function in YOLOv5s. The calculation formula for Focal-EIoU is as follows:


$$\begin{array}{c} L_{EIoU}=L_{IoU}+L_{dis}+L_{asp}=1-IoU\;+\frac{\rho^{\text{2}}(b,b^{gt})}{c^{\text{2}}}+\frac{\rho^{\text{2}}(w,w^{gt})}{C_{w}^{\text{2}}\;}+\frac{\rho^{\text{2}}(h,h^{gt})}{C_{h}^{\text{2}}\;} \end{array}$$ (7)

$$L_{f(x)}=\left\{ \begin{array}{c} -\frac{\alpha x^{2}(2\ln(\beta x)-\text{1})}{\text{4}},0<x\leq 1;1/e\leq \beta \leq 1\\ -\alpha \ln(\beta )x+C,x>1;1/e\leq \beta \leq 1 \end{array}\right.$$ (8)

$$L_{Focal-EIoU}=IoU^{\gamma }L_{EIoU}$$ (9)

In the formula, the variable *χ* signifies the disparity between the actual and predicted values, while *e* represents the mathematical constant. The parameter *β* governs the curvature of the curve, and *C* stands as a constant within the equation. Additionally, the parameter *γ* plays a role in controlling the extent to which outlier values are suppressed.

The Focal-EIoU loss function is particularly suitable for dense pedestrian scenarios and is compatible with the YOLOv5s algorithm. Integrating it into YOLOv5s can improve the model’s detection accuracy and enhance its robustness.

#### 3.1.2. Improvement in small object detection.

One of the reasons for the poor performance of dense occluded pedestrian detection is the small size of small target samples. Due to the high downsampling rate of YOLOv5s, it is difficult for deep feature maps to capture the feature information of small targets. Although the original YOLOv5s model has detection layers with grid sizes of 80 × 80, 40 × 40, and 20 × 20, enabling multi-scale detection, it still suffers from missed and false detections when detecting dense and occluded small pedestrian targets. To address this issue, we propose adding a 160 × 160 SO (Small Object) detection layer to the Neck module. By improving the FPN combined with PAN operations, the shallow and deep feature maps are concatenated for detection, thereby enhancing the model’s detection capability.

**Fig 3:**
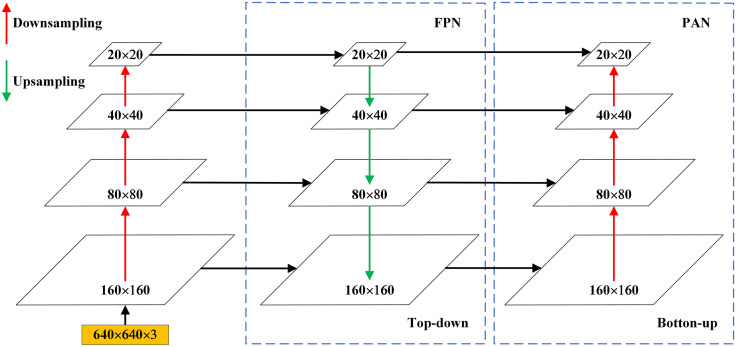
Improved FPN combined with PAN structure.

As shown in Fig 2, after the 18th layer, upsampling and other operations are applied to further enlarge the feature map. At the 21st layer, a 160 × 160 feature map collected from earlier layers is fused with the second-layer feature map from the backbone network. This fusion results in larger feature maps, which improve small object detection performance. The newly added 160 × 160 detection feature map is used to detect objects larger than 4 × 4 pixels. This method is simple and effective, improving the model’s ability to detect objects at different sizes and resolutions in images. The improved FPN combined with PAN structure is shown in Fig 3.

#### 3.1.3. Introduction of the multi-head self-attention mechanism.

The Multi-Head Self-Attention (MHSA) mechanism originates from the Transformer [30] architecture. By constructing a globally correlated attention weight matrix, it enables global modeling of input features, thereby capturing global information more effectively. Additionally, each attention head in MHSA can be computed independently and supports parallel processing, improving the efficiency of model training and inference.

**Fig 4:**
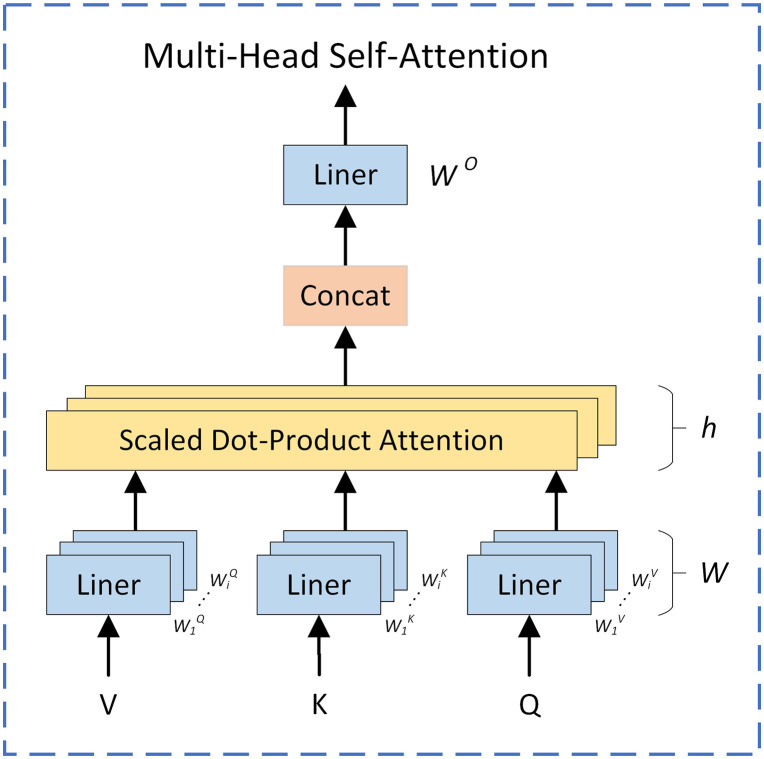
Schematic diagram of MHSA mechanism.

In the YOLOv5s network model, convolution operations are typically local, focusing mainly on specific regions of the input sequence and failing to directly capture global contextual information. This limitation results in insufficient semantic information integration in dense occlusion pedestrian detection tasks. To address this issue, this paper introduces the MHSA mechanism at the end of the YOLOv5s backbone network, allowing the model to perform global self-attention computation on low-resolution feature maps. This helps reduce redundant computations in shallow feature maps, enabling more efficient processing and integration of semantic information related to small-scale targets in densely occluded scenarios. Consequently, the model’s detection performance and generalization capability in dense occlusion pedestrian detection tasks are significantly improved. The structural diagram of the MHSA mechanism is shown in Fig 4.

The computational formula for the MHSA mechanism is as follows:


$$attention(Q,K,V)=softmax(QK^{T}/\sqrt{d_{k}})V$$ (10)

$$head_{i}=attention(Q{W_{i}}^{Q},K{W_{i}}^{K},V{W_{i}}^{V})$$ (11)

$$multihead(Q,K,V)=concat(head_{1},...,head_{h})W^{O}$$ (12)

Here, *Q* represents the query set, *K* represents the key set, *V* represents the value set, *h* represents the number of self-attention heads, *head*_*h*_ represents the computation of the h-th self-attention head, and *W*^*o*^ represents the output weight.

### 3.2. The DeepSORT tracking algorithm

DeepSORT is a target tracking algorithm capable of continuously tracking objects in a video sequence. The main process of the DeepSORT algorithm builds upon the SORT algorithm by incorporating appearance information. It utilizes models from the pedestrian re-identification (ReID) domain to extract features, estimates the tracking target state through Kalman filtering, associates the current frame’s detected targets with the tracking targets from the previous frame using the Hungarian algorithm, and finally updates the target states in real-time using Kalman filtering.

#### 3.2.1. Incorporating the lightweight ShuffleNetV2 network.

**Fig 5:**
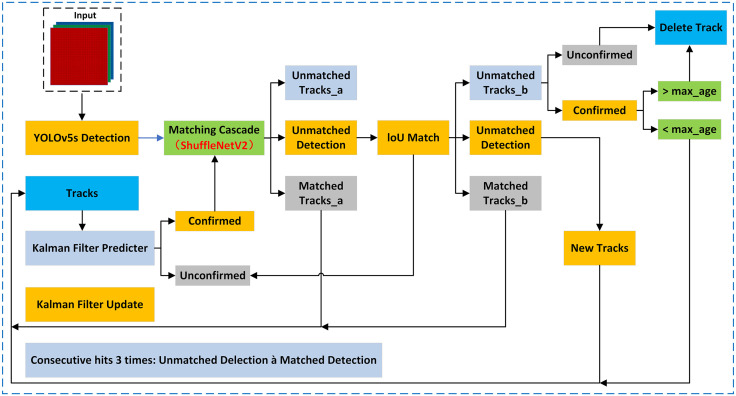
Improved YOLOv5s-DeepSORT network structure.

ShuffleNetV2 [31] is a lightweight convolutional neural network structure that achieves information exchange within network layers through group convolution and channel shuffling. It is used for feature extraction and correlation matching, enhancing feature extraction capabilities while keeping the model lightweight. YOLOv5s is a deep learning-based object detection model with high detection accuracy and fast inference speed. YOLOv5s-DeepSORT combines the YOLOv5s object detection model with the DeepSORT object tracking algorithm. This integration involves replacing the appearance feature extraction network in DeepSORT with the lightweight ShuffleNetV2 network, as illustrated in Fig 5. This approach reduces the overall model complexity and parameter count, improving computational efficiency while maintaining high accuracy. By utilizing features extracted with ShuffleNetV2 for correlation matching, there is a significant improvement in the ID switch metric of the tracking model, a slight increase in tracking accuracy, and the provision of real-time object tracking capabilities.

## 4. Experiments

Under the same experimental environment and training conditions, this paper conducts improvement experiments on the YOLOv5s and DeepSORT algorithms, followed by a comparison and result analysis.

### 4.1. CrowdHuman dataset

The following is a comparison of commonly used pedestrian detection datasets, including Caltech, KITTI, CityPersons, COCOPersons, and CrowdHuman [32]. The comparison results are shown in Table 1. CrowdHuman has 15,000 images in the training set, 5,000 images in the test set, and 4,370 images in the validation set. Compared to other pedestrian detection datasets, CrowdHuman has a larger scale, better representing real-world scenarios. It includes various changes such as different scales, poses, occlusions, densities, and lighting, making it more diverse and challenging. In this study, YOLOv5s experiments used 15,000 images from the CrowdHuman dataset as the training set, divided into a training set: validation set: test set ratio of 6:2:2.

**Table 1:** Volume, density and diversity of different human detection training datasets.

	Caltech	KITTI	CityPersons	COCOPersons	CrowdHuman
# images	42, 782	3, 712	2, 975	64, 115	15, 000
# persons	13, 674	2, 322	19, 238	257, 252	339, 565
# ignore regions	50, 363	45	6, 768	5, 206	99, 227
# person/image	0.32	0.63	6.47	4.01	22.64
# unique persons	1, 273	< 2, 322	19, 238	257, 252	339, 565

### 4.2. Market1501 dataset

The Market1501 [33] dataset was collected and publicly released in 2015 on the campus of Tsinghua University. It includes 32,668 labeled images of 1,501 pedestrians captured from 6 cameras, comprising 5 high-definition cameras and 1 low-definition camera. The images are of size 64 × 128, featuring pedestrians in various poses and perspectives. Each pedestrian appears multiple times across different cameras. The training set consists of 12,936 images of 751 different pedestrians, while the test set includes 19,732 images of 750 different identities. In this study, the DeepSORT appearance feature extraction model was experimentally retrained on this dataset to enable it to extract highly discriminative pedestrian appearance information.

### 4.3. Experimental results and analysis

#### 4.3.1. Model training.

The hardware configuration used in the experiment includes an AMD 3990x CPU, NVIDIA RTX3090 24GB GPU, and 64GB of memory. The operating system is Ubuntu 18.04.6 LTS, and the experiment is conducted using the PyTorch framework. For YOLOv5s, the hyperparameters are set as follows: batch size is 64, epochs is 300, initial learning rate is 0.01, weight decay coefficient is 0.0005, and the SGD optimization algorithm is employed. For DeepSORT, the hyperparameters are set as follows: batch size is 64, epochs is 40, initial learning rate is 0.1, weight decay coefficient is 0.0005, and the SGD optimization algorithm is used.

#### 4.3.2. Evaluation metrics.

This article uses precision (P), recall (R), average precision (AP), and mean average precision (mAP) as the evaluation metrics for the YOLOv5s model. The formulas for calculating these performance metrics are as follows:


$$AP=\int_{0}^{1}PdR$$ (13)

$$mAP=\frac{\sum_{i=1}^{N}AP_{i}}{N}$$ (14)

$$Precision=\frac{TP}{TP+FP}$$ (15)

$$Recall=\frac{TP}{TP+FN}$$ (16)

$$F1=2\times \frac{P\times R}{P+R}$$ (17)

Where AP is the area under the Precision-Recall curve; TP is the number of target boxes with IoU ≥ threshold; FP is the number of target boxes with IoU < threshold; FN is the number of missed targets. The experimental results for YOLOv5s are shown in Table 2.

**Table 2:** Performance Comparison of Different Improvement Methods.

Models	Focal-EIoU	SO	MHSA	P	R	F1	mAP0.5	mAP0.5:0.95	Params	FLOPs
YOLOv5s	×	×	×	0.821	0.666	0.735	0.764	0.458	7.05M	16.3G
Improvement 1	√	×	×	0.821	0.688	0.749	0.778	0.467	7.05M	16.3G
Improvement 2	√	√	×	0.814	0.715	0.761	0.801	0.492	7.71M	27.3G
Improvement 3	√	√	√	0.821	0.715	0.764	0.808	0.497	8.50M	27.9G

The evaluation metrics used in the YOLOv5s-DeepSORT experiment [34] are as follows:

MOTA [35]: Multiple Object Tracking Accuracy. This metric considers three types of errors: false positives, missed targets, and identity switches.

MOTP: Multiple Object Tracking Precision. This metric summarizes the overall tracking precision by calculating the overlap between the true bounding boxes and predicted locations.

IDF1 [35]: ID F1 score. The ratio of correctly identified detections to the average of the true positives and the total number of detections.

IDs: Total number of identity switches.

ML: Mostly Lost targets. The proportion of lost target trajectories does not exceed 20% of the total ground truth trajectories.

MT: Mostly Tracked targets. The proportion of correctly tracked trajectories is not less than 80% of the total ground truth trajectories.

FP: Total number of false positives.

FN: Total number of missed targets.

We evaluated the performance of the improved DeepSORT and improved YOLOv5s-DeepSORT on the MOT16 [36] challenge sequences 02, 04, 05, 09, 10, 11, and 13, and compared them with the original algorithms. The experimental results are shown in Tables 3 and 4, respectively.

**Table 3:** Comparison of Tracking Performance Among Different Improvement Methods with YOLOv5s (↓represents “Lower is better”, ↑ represents “Higher is better”).

Evaluation metrics	DeepSORT	DeepSORT-ShuffleNetV2 (Improved DeepSORT)
MOTA(↑)	47.4%	47.5%
MOTP(↑)	76.8%	76.9%
IDF1(↑)	49.8%	55.6%
IDs(↓)	506	395
ML(↓)	29.4%	31.9%
MT(↑)	25.9%	24.4%
FP(↓)	8527	7587
FN(↓)	49034	50020
Size(↓)	41.6MB	8.3MB
Params(↓)	11.49M	2.02M
FLOPs(↓)	1.13G	0.05G

**Table 4:** Comparison of detection performance of different detection methods.

Methods	mAP0.5	Params	FLOPs	Detection time
Faster-RCNN-ResNet50	0.677	41.35M	91.0G	16.7ms
SSD512	0.534	24.39M	87.72G	15.4ms
YOLOv7-tiny	0.778	6.01M	13.0G	5.8ms
YOLOv8s	0.800	10.64M	28.6G	6.8ms
YOLOv5s	0.764	7.05M	16.3G	7.4ms
YOLOv5s+Focal-EIoU+SO+MHSA (Ours)	0.808	8.50M	27.9G	8.9ms

#### 4.3.3. Ablation experiment.

The comparison of detection performance for different YOLOv5s improvement methods is shown in Table 2. It can be observed that Improvement 1, which replaces the original CIoU loss function with the Focal-EIoU loss function, results in a 1.4% and 0.9% increase in mAP0.5 and mAP0.5:0.95, respectively, while keeping the model’s parameter size and computational cost unchanged. Improvement 2, based on Improvement 1, adds an SO small object detection layer, which increases the model’s parameter size and computational complexity by 0.66M and 11G, respectively, but improves mAP0.5 and mAP0.5:0.95 by 2.3% and 2.5%, respectively. Improvement 3, based on Improvement 2, introduces the MHSA mechanism, which increases the model’s parameter size and computational complexity by 0.79M and 0.6G, respectively, but further improves mAP0.5 and mAP0.5:0.95 by 0.7% and 0.5%, respectively, demonstrating the effectiveness of the improved model’s detection capabilities.

As shown in Table 3, DeepSORT-ShuffleNetV2 is the improved version of DeepSORT, with the number of parameters and computational complexity reduced to 2.02M and 0.05G, respectively, compared to the original algorithm, which saw reductions of 9.47M and 1.97G. MOTA and MOTP also show slight improvements. The model size has decreased from 41.6MB in the original algorithm to 8.3MB in the improved version, shrinking to 20% of the original size, significantly reducing the computational load and effectively demonstrating the efficacy of the model’s lightweight design.

#### 4.3.4. Comparative experiment.

To verify the superiority of the improved algorithm, this paper compares the improved YOLOv5s model with mainstream object detection models, including Faster-RCNN-ResNet50, SSD512, YOLOv7-tiny, and YOLOv8s. As shown in Table 4, the improved YOLOv5s algorithm achieved an mAP0.5 of 0.808, which is a 4.4% improvement over the original mAP0.5 of 0.764. The inference time for a single image is only 8.9ms, demonstrating a clear advantage over other mainstream object detection models, effectively proving the rationality and effectiveness of the proposed improvements.

As shown in Table 5, the improved YOLOv5s-DeepSORT algorithm achieves MOTA, MOTP, and IDF1 of 50.7%, 77.3%, and 58.4%, respectively, representing improvements of 3.3%, 0.5%, and 8.6% compared to the original YOLOv5s-DeepSORT algorithm. In addition, the number of pedestrian IDs is reduced by 57 times, which is an 11.3% reduction compared to the original algorithm. The experimental results demonstrate that the improved YOLOv5s-DeepSORT algorithm effectively addresses the problem of missed and false detections of densely occluded pedestrians on traffic roads to some extent, better meeting the requirements of pedestrian detection and tracking tasks.

**Table 5:** Comparison of tracking performance of different methods.

Evaluation metrics	YOLOv5s-DeepSORT	Improved YOLOv5s-DeepSORT (Ours)
MOTA(↑)	47.4%	50.7%
MOTP(↑)	76.8%	77.3%
IDF1(↑)	49.8%	58.4%
IDs(↓)	506	449
ML(↓)	29.4%	25.3%
MT(↑)	25.9%	25.3%
FP(↓)	8527	8097
FN(↓)	49034	45900

## 5. Conclusion

This paper addresses the pedestrian detection issues in dense occlusion and small targets by improving the loss function, neck structure, and backbone network of the YOLOv5s model. Additionally, lightweight modifications were made to the feature extraction network of the DeepSORT model. The improved YOLOv5s-DeepSORT algorithm significantly enhances the accuracy of pedestrian detection and tracking, reduces false positives and false negatives, and lowers the identity switch error rate, making the pedestrian tracking process more efficient. Future research can explore more efficient lightweight network structures to improve the real-time performance of pedestrian detection and tracking algorithms, thereby better adapting to various application scenarios.
